# The Different Faces of Religion in Therapy: An Exploratory Qualitative Study of a Religion-Based Therapeutic Community for Addiction Recovery in Israel

**DOI:** 10.1007/s10943-024-02152-y

**Published:** 2024-10-13

**Authors:** Michal Pagis, Ayala Elbaz, Yitzhak Ben Yair

**Affiliations:** 1 Department of Sociology and Anthropology, Bar Ilan University, 5290002 Ramat Gan, Israel; 2 Department of Criminology, Bar Ilan University, 5290002 Ramat Gan, Israel; 3 Department of Behavioral Science, Zefat Academic College, 11 Jerusalem St, Zefat, Israel

**Keywords:** Religion, Therapy, Addiction, Judaism, Mental Health

## Abstract

This article examines the impact of the integration of religion and psychological treatment in a religion-based therapeutic community for persons in recovery from addiction in Israel. Based on an exploratory qualitative study that includes participant observation in a Jewish forgiveness therapy training course and in-depth interviews with counselors working in the community, we identify three themes that characterize the therapeutic process. First, religion emerges as a challenge in therapy, one that should be addressed with sensitivity. Second, religion can be turned into a spiritual and cultural resource in the recovery process, with a cautious and sensitive approach. Third, religion can offer a remedial experience, without necessarily involving an intensification of faith. We offer principles that can help guide decisions regarding the integration of religion into therapy, with a focus on addiction treatment oriented to clients with a religious background.

## Introduction

While much has been written about the benefits of religion as a therapeutic resource, research has also indicated that integrating religion and therapy can be challenging and even antitherapeutic (Williams, 2020). Religion-based therapeutic communities (TCs) for addiction treatment are abstinence-based long-term residential environments that integrate psychological therapy with religious practices such as the celebration of holidays and the performance of religious activities (De Leon et al., 2015). Other programs for addiction treatment that involve religion or spirituality, such as the twelve-step, are not tailored to clients with a specific religious background and the integration of religion into therapy is mainly limited to reliance on a higher power. In contrast, religion-based TCs, relatively overlooked in previous research (McBride et al., 2018), are based on a holistic model that integrates various dimensions of religion and serve as an ideal site for examining religion in addiction therapy.

Previous research on religion-based TCs has demonstrated ethical and practical problems related to pressure to intensify faith (Jayne & Williams, 2020; Zigon, 2011). This study, which focuses on a Jewish TC in Israel, found that counselors avoid faith-related pressure, manage the challenging faces of religion, and turn religion into a spiritual and cultural resource that can lead to a remedial experience. The implications of these findings, we suggest, are not limited to the TC model, but can enhance our understanding of the beneficial integration of religion and therapy in other addiction-related therapeutic venues.

## Religion in Therapy: Resource or Obstacle?

Research on the impact of religion in therapy reveals two contrasting effects. On the one hand, religious beliefs can be a valuable asset in the therapeutic process. On the other hand, they can sometimes create obstacles to effective treatment.

### Religion as a Resource in Therapy

According to Pargament et al. (1998), religion may play a significant role in providing individuals with a sense of meaning, purpose, and hope, which in turn may contribute to psychological well-being and resilience. Positive religious coping, such as looking to God for strength and support, is associated with enhanced psychological well-being; conversely, negative religious coping, such as blaming God, is linked to higher levels of depressive symptoms (Harrison et al., 2001; Pargament et al., 1998). These findings are further supported by a meta-analysis, which provides quantification of the strength of these relationships (Ano & Vasconcelles, 2005).

The integration of religious and spiritual beliefs into therapeutic approaches has become a significant advancement (Elkonin et al., 2014). Researchers stress the importance of assessing clients' religious and spiritual beliefs in clinical evaluations, as it enables therapists to incorporate religion as a resource for healing (Rose et al., 2001). Martinez et al. (2007) found that clients viewed various therapist religious interventions such as referencing scripture, teaching spiritual concepts, encouraging forgiveness and utilizing religious community resources as appropriate and beneficial. Studies have shown that religiously integrated therapies can effectively address mental health concerns like depression, anxiety, and substance abuse (Captari et al., 2018). Recent decades have seen the integration of religious and spiritual values into various therapeutic models, including spiritually integrated psychotherapy (Richards & Bergin, 1997), religiously integrated cognitive–behavioral therapy (Sabki et al., 2019), and rehabilitation of offenders using religious scriptures (Ben Yair, 2021).

Studies have shown the value of spirituality and religion in the prevention, treatment of, and recovery from substance use (Beraldo et al., 2019; Travis et al., 2021; Unterrainer et al., 2013). In this context, religion and spirituality are associated with positive outcomes such as improved coping, lower levels of anxiety, resilience, optimism, and social support (Pardini et al., 2000). Integrating faith-based interventions into addiction treatment increases the motivation for drug abstinence (Beitel et al., 2007). The early twentieth century witnessed the emergence of a number of addiction recovery programs which integrate medicine, psychology, and religion (White, 2000). While early programs, such as the twelve-step, were based on a Christian framework that became more religiously diverse over time, contemporary programs have shifted to a more universal understanding of spirituality (Snodgrass et al., 2023). An example of the latter is spirituality-based addiction counseling (İşbilen & Mehmedoğlu, 2022), which is suited to a broad array of cultures and faiths.

### Religion as an Obstacle in Therapy

Alongside the growing consensus around the benefits of integrating religion and spirituality into therapy, studies have also shown that such integration may not always be beneficial (Cashwell & Swindle, 2018; Fallot, 2007). One major challenge is that differences in theology or spiritual beliefs might have a considerable impact on what clients expect, hope for, and need in therapeutic situations (Post & Wade, 2009). Misalignment between the religious worldviews of the client and the counselor can cause distress as clients expect their counselors to be open to their religious views and to respect their values and beliefs (Latzer et al., 2019). Counselors need to strive to understand the values of the specific culture in the service of their religious patients' recoveries (Johnson & Hayes, 2003; Latzer et al., 2019).

A second challenge arises when negative experiences in a religious community lead to negative attitudes toward religion and God (Pargament, 1998; Zarzycka, 2016). This challenge is especially common in cases in which clients experienced abuse at the hands of a religious leader or a religious community (Cashwell & Swindle, 2018). “Religious strain” (Fallot, 2007, p. 264) manifested in conflict with God and with the religious community, struggles with disbelief, and self-blame regarding lack of virtue, may all lead to clients’ ambivalence and even resistance regarding the integration of religion into the therapeutic process (Exline et al., 1999; Exline, 2019).

Studies on addiction have shown that integrating religion and spirituality into addiction treatment is complex and may act as a “double-edged sword” (DiClemente, 2013, p.1260). In some cases, spirituality and religion serve as risk factors, as drug use may be connected to rebellion against strict religious communities or to a search for spiritual enlightenment (DiClemente, 2013; Room, 2013). In addition, anger toward God may be linked to addiction (Stauner et al., 2019). Individuals may have had past traumatic experiences related to religion, and thus, the introduction of religion into addiction treatment may lead to varied reactions, from openness and engagement to ambivalence and doubt (Diclemente, 2013). Last, strict religiosity appears to be related to more negative views toward addictions (Weinandy & Grubbs, 2021), and individuals may find themselves confronting “divine struggles” (Currier et al., 2020, p. 324). For example, Christian communities in Brazil enforce a view of drug use as a personal problem linked to sin and the meddling of evil forces (Bardi & Garcia, 2022). Such perspectives may lead to the use of religious interventions such as spiritual purification and confession, which in some cases are effective, but may also lead to increased internal conflicts and self-blame.

Overall, the research indicates that the integration of religion into therapy holds promise but also poses challenges. A deeper investigation is required to understand how to manage the more challenging faces of religion in therapy, and under what conditions and contexts such integration can be beneficial. One important context, relatively overlooked in the literature, is religion-based therapeutic communities (McBride et al., 2018). These communities are based on a holistic model that integrates various aspects of religion that go beyond the general principle of faith. The holistic approach makes these communities an ideal site for examining religion's varied faces in therapy.

## Religion-based Therapeutic Communities for Addiction Treatment

Therapeutic communities (TCs) for addiction treatment are long-term abstinence-based residential programs that align treatment stages with increasing personal and social responsibility (De Leon, 2000). This holistic approach aims for full recovery and a return to a socially productive lifestyle (De Leon et al., 2015). Most therapeutic communities contrast with harm reduction methods, which view addiction as a chronic illness and focus on minimizing negative consequences while allowing continued substance use. The TC model emphasizes complete rehabilitation rather than managing ongoing addiction.

Therapeutic communities are based on the notion of community-as-method as “the purposive use of the community to teach individuals to use the community to change themselves” (De Leon et al., 2015, p. 62). In TCs, various treatment techniques are integrated simultaneously, including psychological therapy, twelve-step, and animal-assisted therapy. The TC model emerged in Christian religious organizations in Europe and can be traced back to the nineteenth century. Today, however, TCs are predominantly non-religious (McBride et al., 2018) and designed to serve clients with diverse religious and non-religious backgrounds.

While such a universal approach has benefits for a more global application, it is not tailored for clients with religious backgrounds. Religion-based TCs adapt the traditional model for clients with specific religious backgrounds. These communities incorporate religious observances, holidays, and practices like prayer, fasting, and scripture reading into the community-as-method approach (De Leon et al.,, 2015). The collective practice of religious rituals and the shared religious beliefs reinforce community commitment, while religious principles and ethics are recruited to support abstinence (Bardi & Garcia, 2022).

Most research on TCs has been conducted in Europe and the US, and focuses on non-religious communities (McBride et al., 2018). The few studies on religion-based TCs show that in this context as well, religion is both a resource and a challenge. Religion-based TCs help clients to produce a new socially functioning identity in a supportive religious community (Bardi & Garcia, 2022; Hansen, 2005; Jozaghi et al., 2016; Rashid et al., 2014). When narrating their stories of recovery from addiction, individuals tend to weave together healing and an intensification of religious faith (Sremac & Ganzevoort, 2013). For example, in a Sufi TC located near Jericho, clients “appear to be radically transformed” from agnosticism or nominal adherence to Islam at the beginning of the program, to strict religious adherence at the end (Al-Krenawi & Graham, 1997, p. 384).

Yet, this emphasis on religious adherence can be a double-edged sword (Bardi & Garcia, 2022). Studies show that religion-based TCs sometimes pressure clients to participate in religious activities and increase their faith or even convert (Jayne & Williams, 2020; Williams, 2020; Zigon, 2011). Research on Christian TCs demonstrates that many of these communities aim to bring people closer to Christ, following the theological rehabilitation principle that “it is Christ who sets them free from drugs” (Mikeshin, 2020, p. 53). Such integration of recovery with intensification of religious faith holds the potential to become antitherapeutic (Williams, 2020), especially when compulsory faith-based activities, such as prayer or confession, are experienced by clients as oppressive (McBride et al., 2018).

These studies, while highlighting important dimensions of religion in the addiction recovery process, are relatively limited in scope. There is a need for deeper understanding of how religion is used in such communities and how religion-based activities interact with the community-as-method approach. The purpose of this study is to explore how counselors manage the challenges of integrating religion into the recovery process and what strategies help them to balance the beneficial and challenging aspects of religion in therapy.

## Retorno Religion-based Therapeutic Community

Founded in 1989, Retorno is a long-term residential Judaism-based TC located on the outskirts of Bet Shemesh, a city in central Israel. It is based on the core TC principles of mutual aid and self-help (Ronel et al., 2015) and is aimed at people who struggle with substance abuse, compulsive gambling, or sex addiction. Retorno was founded by professionals who employ the services of recovered individuals as counselors alongside the professional staff.

While Retorno TC accepts all clients, most come from Orthodox and ultra-Orthodox Jewish families. In Israel, “religious” typically refers to Orthodox Judaism, as liberal Jewish movements are less common and attract a more secular audience (Pagis et al., 2018). Orthodox Judaism is culturally conservative, with ultra-Orthodox communities (13% of Israeli Jews) often resisting external influences and seeking separation from broader society (Sivan, 1995). The conservative and communal nature of Orthodox groups pose challenges to addiction treatment, as these communities often view addictive behaviors as contradicting religious values and tend to be suspicious of secular therapeutic approaches. Retorno's approach is tailored to accommodate the cultural and religious needs of this particular demographic, filling a crucial gap in addiction treatment services in Israel.

In Israel, addiction treatment primarily focuses on abstinence, with harm reduction approaches being less common and often controversial (Bonny-Noach, 2019). This is particularly true in Jewish Orthodox communities, which are typically conservative, preferring treatments aimed at full recovery. Retorno is unique in offering a Jewish religious environment and community that is in resonance with the Orthodox religious Jewish background of most residents.

Retorno integrates Jewish principles and practices into its treatment approach in a number of ways. First, clients are encouraged to participate in Jewish traditions such as Sabbath candle-lighting and observance of the kosher dietary laws. Additionally, the program offers Jewish study sessions that explore the ethical and moral teachings of Judaism, with a focus on how these can be applied to overcoming addiction. The program emphasizes the importance of community and social support, encouraging clients to participate in group activities and events. Retorno's treatment program integrates twelve-step principles, such as faith in a higher power and personal responsibility for recovery. Additionally, it offers a range of therapeutic approaches, including dynamic psychotherapy and music therapy.

Retorno addresses the problem of addiction at its various levels, through several units: an education center for the prevention of addiction, detox centers, a therapeutic community, and an outpatient clinic. The TC admits individuals aged seventeen and older. The young adults (ages 17–25) are physically separated from the older adults and attend school in the community center. Due to the program’s religious character, men and women are treated separately in all the therapeutic units.

## Methods

Data were gathered in two steps. The first included ethnographic observations in a training course on Jewish forgiveness therapy (Ben Yair et al., 2024), delivered at Retorno in 2021. The second included in-depth interviews with 15 counselors who work at Retorno.

### Ethnographic Observations: Context and Participants

The first and the second authors observed a training course on Jewish forgiveness therapy delivered in Retorno and documented the dialogues and interactions in an ethnographic diary. An adaptation of Enright’s (2015) forgiveness therapy, Jewish forgiveness therapy integrates Jewish religious nuances and is informed by the assumption that past trauma, particularly within the family system, can lead to drug abuse and criminal behavior (Hargrave & Zasowski, 2016). The training course included twelve meetings, two (the first and the last) conducted in person and ten through Zoom. Participants included twelve counselors, all working at Retorno: five social workers, five twelve-step sponsors who also work as group leaders, and two chaplains (rabbis).

### Interviews: Recruitment, Participants, and Method

Fifteen in-depth, face-to-face interviews were conducted with counselors who work at Retorno, including social workers, occupational therapists, twelve-step sponsors, and chaplains (Rabbis) (See Table 1 for demographic characteristics). Ten of the interviewees also participated in the Jewish forgiveness therapy training. We approached counselors individually, asking if they were willing to be interviewed. While we attempted to recruit participants with diverse demographic characteristics, the sample is not representative.

**Table 1:** Participants’ demographic characteristics (N = 15)

Demographics		N (%)
Gender	Female	8 (53%)
	Male	7 (47%)
Religious Self-Identification	Jewish Religious	11 (73%)
	Jewish Secular	4 (27%)
Occupation	Social Worker	6 (40%)
	12 Steps Sponsor	5 (33%)
	Occupational/Art Therapist	2 (13%)
	Chaplain (Rabbi)	2 (13%)
Age	20–30	1 (7%)
	30–40	3 (20%)
	40–50	6 (40%)
	50–60	2 (13%)
	60–70	3 (20%)
Education	High school diploma	2 (13%)
	BA	2 (13%)
	MA	8 (53%)
	Ph.D.	1 (7%)
	Post-high school Yeshiva	2 (13%)

The research method involved conducting in-depth interviews using a semi-structured format (Corbin & Strauss, 2015; Jamshed, 2014). We asked open-ended questions, allowing participants to respond freely and elaborately. The interview guide we developed focused on two main areas: 1. participants' work experiences at Retorno and 2. participants' perceptions and experiences regarding the role of religion within Retorno. This approach provided a consistent framework for all interviews while still allowing for flexibility in participants' responses. Interviews lasted between thirty minutes and one hour and were recorded and transcribed.

### Ethical Considerations

To avoid identification, each participant was assigned a number (P1-P15) and is identified by that number in the text. The demographic table summarizes participant characteristics as a group, rather than listing details for each individual. This approach maintains participant anonymity. In addition, we use the general title counselor to denote all interviewees, regardless of their specific care profession. Informed consent was obtained from all participants in the study. This study was approved by the Institutional Review Board at Zefat Academic College (no. A 27–2021).

### Data Analysis

Data were analyzed following a phenomenological, abductive approach (Tavory & Timmermans, 2014). Initially, we used the code “religion and spirituality” to identify meaningful sections of the transcriptions that dealt with religion in therapy. In the second phase of analysis, this code was further deconstructed into subthemes as described in the findings section. All quotes in the text are verbatim, translated from Hebrew to English, with the relevant participant number (e.g., P1) appearing at the end of each quote.

## Results

**Fig. 1:**
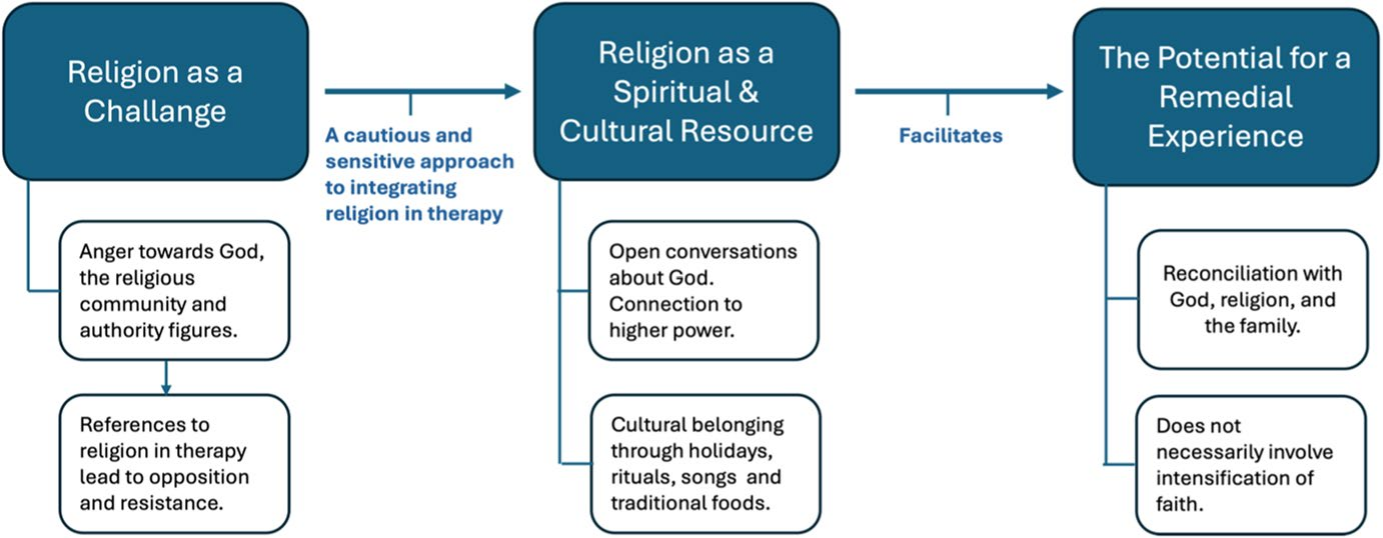
Themes and subthemes

Our analysis revealed three themes that characterize the therapeutic process at Retorno (Fig. 1). First, we demonstrate that religion emerges as a challenge in therapy, one that should be dealt with sensitively and stealth. Second, we show that a cautious and balanced approach can turn religion into a spiritual and cultural resource in the recovery process. Third, we show that religion can offer a remedial experience, without necessarily an intensification of religious faith.

### Religion as a Challenge

While counselors acknowledged the beneficial aspects of religion in therapy, they agreed that at the beginning of the therapeutic process, religion usually appears as an obstacle. This is because religion tends to “sit” on the place of the trauma that led the clients to the path of addiction. The counselors spoke of anger that clients carry toward religion, the religious community, and God. As one counselor described it:

[Some think] that he [a client] left the [religious] path because he found drugs and started using. But actually it is the other way around. Because he needed to leave the religious place, and it is not easy to leave a place, you leave a place because you are not happy, because they will not accept you and more, and this creates pain that leads to [drug] use. So often, there is a lot of anger toward the parents, toward God, toward the Torah (P8).

In the close-knit Jewish Orthodox community, religion, authority, and family are intertwined. Anger toward parents or community often manifests as anger toward religion itself. For example, counselors at Retorno noted that some women express anger toward the religious community for inadequate sexual education or toward parents whose large families (common in ultra-Orthodox communities) led to neglect. This anger frequently extends to the broader religious perspective and sometimes to God.

Such anger can turn religion into a challenge in the therapeutic process. The use of religious texts, the encounter with religious authority figures, or the observance of religious law can trigger resentment and opposition. For example, in the women’s community at Retorno, the chaplain [rabbi] has a group talk with the women once a week. While the majority do not object and say that they like the rabbi, the women still express conflicted views regarding these weekly sessions. As one counselor told us: “Some will not say it in front of him, they will say it afterward – I did not listen to him because he reminded me of my uncle, my grandfather, my rabbi” (P12). This male religious figure, who reminds the women of the religious men in their communities of origin, becomes a target of objection and resistance.

Moreover, reference to religion is sometimes interpreted by the clients as a reference to power and authority, as one counselor stated: “I don’t want to do it [use religious sources] because some girls left religion because they were hurt. I don’t want them to think I am wielding power over them” (P9). For these girls, explained the counselor, religion symbolizes power and authority over their bodies and identities, a power that harmed them in the past.

The debatable place of religious texts in therapy was evident in the training course on Jewish forgiveness therapy, where we conducted ethnographic observations. In one of the lessons, a counselor who himself recovered from substance abuse disorder and grew up in an Orthodox Jewish religious community, shared his response to the texts introduced in the course. These excerpts from the Jewish Talmud or from the book *Orchot Tzaddikim*, were familiar to him, as he had studied them at the yeshiva [Jewish academy for Talmudic study]. However, instead of connecting him to forgiveness, they deter him from connectedness. As he stated, “I know these texts, they don’t add something new, I know where it starts and where it is going to…” (P7). The repetitive reading of these texts in the yeshiva stripped them of meaning. As a result, these texts now carry negative connotations and hinder, rather than support, his healing process.

The counselors stated that it is crucial to bear in mind that activities, texts, or discussions perceived as connected to Judaism might elicit resistance and should be introduced with caution. For example, one of the counselors explained how he connects the recovery process to Jewish sources, but then added that when he speaks to the clients “I do not offer the interpretation that it comes from the Torah” (P5). When asked why he keeps these interpretations to himself, he explained that talking about the Torah is a challenge and that, in fact, for the clients, it is sometimes better to have the Torah hidden. Likewise, another counselor explained: “I do not do that [use religion] on an explicit level. I do not use a religious discourse” (P2). Like other counselors, this counselor went on to say that the integration of religion in therapy requires a great deal of sensitivity.

### Religion as a Spiritual and Cultural Resource

Counselors are cautious in their mention of religion, especially at the beginning of the therapeutic process. Nonetheless, they report that they find sensitive and stealth ways to use religion as a spiritual and cultural resource in the therapeutic process. Counselors use the term “spiritual” to describe a religious dimension involving faith in God or a higher power without adherence to a specific religious tradition.

Many clients in Retorno grew up believing in God; thus, their relationship with God is central to their self-identity. This relationship was seen by the counselors as an important part of therapy. As one counselor put it: “We can talk about God … Many times, they won’t talk about their parents, but they will talk about God” (P15). The counselors stressed that in other therapeutic contexts they had encountered outside Retorno, talking about God might be considered unusual. In contrast, at Retorno, conversations about God are seen as appropriate. As expressed by another counselor: “The religious frame [of the community] enables a spiritual conversation. In this context, it is very legitimate to talk about my relation to God as existing and present” (P6). Or as a third counselor said, “I can get God out of the closet,” (P1) hinting to her experience in other therapeutic contexts, where she needed to keep God under wraps.

Notably, our interviewees stressed the importance of keeping conversations about God completely open so that their clients feel free to express anger or doubt toward God, together with seeing God as a role model or ally. Many clients have doubts and struggles regarding religion and God. As one counselor said: “They ask how did I reach this place, where was God? How did this happen to me … these are questions that arrive from a painful place” (P15). These questions, explained counselors, are key for the process of healing, and thus, the invitation of God into the therapeutic process should be done with respect and sensitivity.

Another way to introduce religion as a spiritual resource is to rely on the universal, not specifically Jewish, twelve-step approach to spirituality. A counselor explained that “many clients in this specific community come from Orthodox families where they experienced the religious dimension as something strict. The spiritual element of connection is something that requires a very subtle and sensitive observation” (P2). The shift from strict religion to spirituality requires work, and sometimes it is done more easily when relying on resources that are distant from Jewish connotation. The twelve-step, said the counselors, teach the clients that “any spirituality is good. We need to find a higher power, greater than us, and submit to this force, and it is a spiritual force that offers a solution” (P13).

Besides the positive effect of connecting to God and to a higher power, the counselors stressed the positive role of Judaism as a cultural resource that produces a feeling of belonging to a community, which promotes healing. According to the counselors, various Jewish elements serve as cultural resources, including the daily prayers, the holidays, the dietary laws and the Sabbath. All these produce a sense of familiarity, connection, and belonging. As one counselor put it: “The clients like the prayers, the holidays, the religious time frame. Before each holiday there is a special atmosphere here” (P11). Another counselor described the women's community: “There is a strong connection to Jewish tradition. They really need it. The welcoming of the Sabbath, the festiveness. For example, making challah [Jewish bread] for the Sabbath. It is very significant…” (P3).

Another counselor who works with the men further explained how Retorno can offer cultural belonging even for clients who do not follow the Jewish religious rules:

There are kids [clients] that grew up in a certain way, and their mentality and way of life are different, even if right now they do not keep Torah and Mitzvot [Jewish law] … there are studies that show that kids who leave the Orthodox community commit suicide at higher rates because they cannot connect to secular society in the right way. So this place offers a solution for this, that even though people no longer keep Torah and Mitzvot they still feel more at home, in their safe space (P5).

In this quote, the counselor argues that religion is more than a religious practice or faith. Religion is a mentality, a way of expression, a culture, that offers a feeling of belonging.

The counselors stressed that while the Retorno schedule includes daily prayer, observance of the Sabbath and celebration of the Jewish holidays, the counselors are careful to frame these rules as non-coercive. Thus, keeping to the schedule is understood as crucial, but not justified by religious reasons. As the above counselor continued:

We do not force anyone to observe the Sabbath, to pray … We have the rules of the place. You have to show up for prayer, but you do not have to pray. You can read Harry Potter … we tell them, you can sit here, if you do not want to pray this is yours … we do not force anyone …This [coercion] is what others have done to them in life … This is why they distanced themselves [from the religious community] (P5).

Aware that religious rules may be experienced as coercion, the counseling staff introduces the rules as communal and cultural, and not as religious. This strategy again illustrates the cautious approach taken at Retorno to making explicit reference to religion.

### The Potential for a Remedial Experience

As illustrated above, a sensitive and balanced approach can turn religion into a spiritual and cultural resource in the recovery process without ignoring religion’s challenging face. Such an approach may lead to what counselors refer to as a remedial experience. A remedial experience does not necessarily involve an intensification of religious faith. The counselors all stated that the therapeutic process in Retorno does not aim to strengthen clients’ religious faith or identity. Yet, at the same time, since, for many clients, addiction is connected to disappointment by the religious world, an ideal process of recovery includes some reconciliation with religion.

The community offers a unique opportunity for remedial experiences that can heal experiences of rupture. As one counselor said, “We try to give clients a little taste of a remedial experience. We try to show them that there is a loving God” (P8). Yet, as illustrated above, many clients have doubts and struggles regarding religion and God. This is why counselors are careful not to impose religious doctrines, discourse, or faith. One put it aptly: “They need to want the remedial experience … this is not something explicit. It is open to those who want to choose ….” (P4). The counselors repeated that “faith is not something we can force on people. It comes from within” (P11). Therefore, counselors in the Retorno community are careful not to explicitly stress reconciliation with the religious world. Such reconciliation is open to those who want it and usually develops only in the later stages of the healing process.

A central factor that facilitates remedial experience is embeddedness in a religious environment that is comforting and not threatening. The celebration of religious holidays creates a special environment in a positive religious setting. Even those who tend “to boot out religion,” (P3) as a few counselors put it, may find themselves praying, or suddenly, after months of resenting God, say that “today God was with me” (P3). These remedial experiences are not necessarily permanent. They represent a step on the way to recovery, and clients frequently remain ambivalent regarding religion. Yet the community still offers a place where, if there is a will, there are opportunities to explore experiences of reconciliation.

One of the counselors, a former Retorno client, described such a remedial process:

I can testify from my own treatment that at least for two-thirds of my therapeutic process I had a lot of anger toward God. I had no connection to the spirit because I had so much hate for what I had to go through all these years. But closer to the end of the treatment, I started to deeply connect to God, I saw things happening to me, and I found peace in my connection with God (P14).

The counselor added that at the beginning of his stay in Retorno he did not actively participate in the prayers: “I sat there, but I wrote letters, I did not pray” (P14). This changed toward the end of his treatment, when a deep connection to God surfaced and he started praying daily. Since leaving Retorno, he no longer prays, but he explained that at that time, the connection to God and the prayers were an important anchor for his healing.

Perhaps the most important aspect of a remedial experience is the connection back to the family. Orthodox Judaism is highly familial and collective. Strict adherence to religious law is often required by the family. At Retorno, clients learn to live in a religious community, and this experience enables them to stay with their families and respect religious rules (even if they do not personally endorse these rules). As one counselor explained, "They may not return to religion, but they do return to the family. The family accepts them. They learn how to respect religious laws not necessarily because the rules are religious, but because they respect the world of the family" (P4). In this way, clients learn to accept and be accepted by their families, a step that is crucial for recovery, especially for younger clients.

## Discussion

This study illustrated that religion in therapy has both negative and positive faces. Religion can serve as a spiritual and cultural resource in therapy (Captari et al., 2018; Travis et al., 2021). Yet, due to the tendency of religion to intersect with trauma (Currier et al., 2020), clinicians use caution in its implementation. Nonetheless, even when religion presents a challenge, it can offer a remedial experience that may appear in later stages of therapy.

Counselors employed various strategies to manage religion's challenging aspects. For example, they limited religious references when they might trigger resentment, allowed clients to express anger toward God, and presented religious practices as community norms rather than laws. Recognizing that some clients struggled with religious figures or discourse due to past experiences, counselors respected their reluctance to engage. These approaches helped mitigate the faith-related pressure issues reported in other religion-based TCs (Jayne & Williams, 2020; Zigon, 2011), creating a more balanced therapeutic environment.

This study illustrated how religion interacts with the community-as-method approach (De Leon et al., 2015; McBride et al., 2018). While previous research on religion in therapy focused on the spiritual aspect of religion (İşbilen & Mehmedoğlu, 2022; Richards & Bergin, 1997), this study illustrated that religion in therapy can also serve as an important cultural resource that enhances community belonging. The notion of cultural religion refers to contexts where religious practice provides communal support and belonging without necessarily involving faith (Zuckerman, 2020). Judaism can be practiced on different levels, and celebrating holidays, referring to Jewish ideas, or preparing Jewish foods, can connect clients to their childhood and create a feeling of belonging (Pagis et al., 2017). Religion-based TCs can use religious rituals and practices in order to produce communal belonging and commitment without the pressure to endorse particular tenets or to follow particular religious laws. A therapeutic community based on a shared religious background can benefit clients even when they have doubts regarding their faith.

The study primarily focuses on counselors' approach to integrating religion in therapy, but we can extrapolate insights about the different systems and layers at play. On the individual level, a sensitive and balanced approach involves allowing personal expression of anger, doubt, or struggles with God and religion, and open spiritual conversations without imposing religious doctrines (Fallot, 2007). At the community level, this approach involves creating a familiar cultural environment through religious traditions, holidays, and dietary practices, providing a supportive religious environment that is comforting rather than threatening, and facilitating a sense of belonging through shared cultural practices (Zuckerman, 2020). In the broader societal context, this approach recognizes the interconnectedness of religion, family, and community, and prepares clients for reintegration into their families and communities, even if they do not fully embrace religious practice.

### Implications for Counseling

The findings of this study may have important treatment implications. First, in religion-based TCs, it is beneficial to have counselors with varied attitudes toward religion and spirituality, ranging from secular to religious. This arrangement will help to ensure that clients can connect with at least one counselor with whom they feel that therapy includes the right amount and right dimensions of religion. Second, in religious TCs as well as in other therapeutic contexts, counselors should bear in mind that relationships toward religion tend to change over time, and thus, it is important that clients know that if they choose to, they have the option to engage more, or less, with religion. This can open up possibilities for a remedial experience. Third, it is beneficial to distinguish between the cultural and the faith-based aspects of religion (Zuckerman, 2020). Rituals, celebrations, songs, or traditional foods can be used in ways that are not necessarily connected to belief but can produce a sense of belonging and community as a base for recovery.

While these implications are especially relevant to TCs, they can also enhance a beneficial and effective integration of religion and therapy in other venues that serve clients with religious backgrounds. A sensitive and balanced approach that includes conversations about God or a higher power, and the use of religious practices as a cultural resource that enhances a sense of belonging, can help clients deal with “religious strain” (Fallot, 2007: 264), heal ruptures, and achieve reconciliation with the religious world.

### Recommendations for Future Research

A study that incorporates interviews with clients from different religious backgrounds and with varying levels of religiosity would add an important perspective to the inquiry (see for example Zigon, 2011). In addition, a comparative study with non-Jewish, religion-based TCs would be a fruitful extension of the research. Finally, further research should examine the aforementioned treatment implications in other addiction-related therapeutic venues that serve clients with religious backgrounds but do not involve long-term residence.

### Limitations

The study is based on in-depth interviews solely with counselors and not with clients.

The number of counselors interviewed is relatively small and is not representative. In addition, the study is also limited to a sample from one religion-based TC in a particular location.

## Conclusion

This study examined the impact of the integration of religion and psychological treatment in a therapeutic community for Jews in recovery from addiction in Israel. Based on interviews with counselors working in the community, this study strengthened the understanding that even when clients have a religious background, the beneficial aspects of integrating religion into therapy cannot be assumed (DiClemente, 2013; Fallot, 2007). A sensitive and balanced approach can turn religion into a spiritual and cultural resource in the recovery process and eventually lead to a remedial experience.
